# Bias in Gene-by-Environment Interaction Effects with Sum Scores; An Application to Well-being Phenotypes

**DOI:** 10.1007/s10519-023-10137-y

**Published:** 2023-03-01

**Authors:** Dirk H.M. Pelt, Inga Schwabe, Meike Bartels

**Affiliations:** 1 Department of Biological Psychology, Vrije Universiteit Amsterdam, Amsterdam, The Netherlands; 2 Amsterdam Public Health Research Institute, Amsterdam University Medical Center, Amsterdam, The Netherlands; 3 Department of Methodology and Statistics, Tilburg University, Tilburg, The Netherlands

**Keywords:** Twin studies, Sum scores, Measurement error, Item Response Theory, Genotype by environment interaction, Well-being

## Abstract

In the current study, we investigated the influence of using skewed sum scores on estimated gene-by-environment interaction effects (GxE) for life satisfaction and happiness with perceived social support. To this end, we analyzed item-level data from a large adult twin sample (*N*s between 3610 and 11,305) of the Netherlands Twin Register. Item response theory (IRT) models were incorporated in unmeasured (univariate) GxE models, and measured GxE models (with social support as moderator). We found that skewness introduced spurious GxE effects, with the largest effect for the most skewed variable (social support). Finally, in the IRT model for life satisfaction, but not for happiness, heritability estimates decreased with higher social support, while this was not observed when analyzing sum scores. Together, our results indicate that IRT can be used to address psychometric issues related to the use of sum scores, especially in the context of GxE, for complex traits like well-being.

**Supplementary Information:**

The online version contains supplementary material available at 10.1007/s10519-023-10137-y.

Broadly, well-being refers to individuals’ evaluation of the positive and negative aspects in their lives. Different conceptualizations and measures of well-being exist, for example, *quality of life*, *satisfaction with life*, *positive affect*, and *happiness* (Layard 2010). Overall, well-being is found to be related to a number of positive outcomes, such as increased mental and physical health, better social relations, and better functioning at school and work (Diener et al. 2018). It is therefore important to find answers to the question: why are some people happier than others? Part of this answer lies in the genetic differences between people. Meta-analyses on the results of twin studies indicate that around 40% of the variance in well-being is due to additive genetic effects (A), with the remainder being due to non-shared environmental effects (E). Evidence for the influence of the shared environment (C) or non-additive genetic effects (D) is limited and inconsistent (Bartels 2015; Nes and Røysamb 2015).

Gene-environment correlations and gene-environment interaction effects have previously been found for well-being (Røysamb et al. 2014). Gene-environment correlation (rGE) occurs when differences in individuals’ genes are related to the exposure to specific environments (Plomin et al. 1977). We speak of gene-environment interaction (GxE) when the effect of an environmental exposure varies depending on an individuals’ genotype, or alternatively, the effect of one’s genetic predispositions depend on the environment in which these are expressed. An environmental exposure that is frequently investigated in these contexts is perceived social support^1^, which typically shows modest positive phenotypic correlations with well-being (Chu et al. 2010; Mann et al. 2019).

Social support has been shown to be moderately heritable (Kendler 1997; Wang et al. 2017; Mann et al. 2019). This in itself is an indication of gene-environment correlation as it shows that differences in exposure to an environment (social support) can be partly explained by genetic differences. In addition, some of the genes responsible for individual differences in social support also influence well-being as indicated by sizeable genetic correlations (Kendler 1997; Wang et al. 2017; Mann et al. 2019; Van De Weijer et al. 2021). This finding may be explained by *reactive* rGE: when individuals are genetically predisposed to being happy (as opposed to being unhappy or depressed), they may be more easy to spend time with and more prone to illicit social support from others when needed (Mann et al. 2019). Happier individuals may also be more prone to seek out social support (*active* rGE).

Social support has also been found to moderate the genetic and environmental effects on well-being (GxE). Nes et al. 2010 for example, showed that the (standardized) proportion of non-shared environmental variance in well-being was larger (i.e., heritability was lower) for married compared to unwed individuals. For married people, genetic predispositions for being (un)satisfied with life may play a smaller role since they enjoy the protection of social support that marriage offers. Unmarried people may be more socially vulnerable resulting in their well-being being more dependent on genetic predispositions. Similar moderating effects of social support (i.e., a smaller standardized proportion of genetic variance at higher support levels) have been found for traits related to well-being, such as depression (Heath et al. 1998; Beam et al. 2017), perceived stress (Beam et al. 2017), and internalizing disorders (South and Krueger 2008).

In Nes et al. 2010, GxE was tested by comparing heritability estimates across different groups (wed vs. unwed). Alternatively, one can model GxE effects in variance component (twin) models (Purcell 2002). One caveat, however, with these approaches is that scaling issues (i.e., skewness) of the phenotypic measures can bias results by introducing spurious GxE effects (Eaves et al. 1977; Molenaar et al. 2012; Molenaar and Dolan 2014; Schwabe and Van den Berg 2014). In well-being research, this is a relevant issue since well-being measures typically show ceiling effects: most people are usually quite satisfied with their lives and therefore the majority of respondents achieve very high scores (and relatively few average or lower scores). In this case, measurement error variance is not homogeneously distributed across the scale as it varies for different levels of the observed measure of the phenotype. Consider two individuals who are different from each other and in the top part of the *latent* well-being distribution. Due to the scale’s properties, they will appear to be more similar in their well-being levels than what their ‘true’ latent well-being values would suggest. Thus, with negatively skewed scores, there appear to be smaller individual differences at the upper (vs. lower) end of the measurement scale. In a variance decomposition this will be captured in the E component (as it also captures measurement error) and therefore result in a lower amount of (raw) E variance for twins with higher scores than for average or low scoring twins. Spurious GxE interactions can therefore be expected since the estimated amount of E depends on the (genotypic) value of the trait.

This spurious GxE effect can appear in the univariate case, i.e., where the environmental variance in a trait depends on the genotypic value of the trait itself (unmeasured GxE; Molenaar et al. 2012; Schwabe and Van Den Berg 2014; Schwabe et al. 2017a), but also extends to cases including a measured moderator (e.g., Schwabe et al. 2017b). Consider social support and well-being, which show moderate to strong phenotypic and genetic correlations, and assume that both phenotypes have skewed sum score distributions with ceiling effects (a likely empirical scenario). In this case, twins at the higher end of the social support scale (i.e., the moderator) will generally score higher on the skewed well-being scale. Due to this skewness, they will (spuriously) appear to be more similar in their well-being than they truly are – as outlined above. Consequently, the results of a twin analysis will spuriously show that (unstandardized) non-shared environmental variance decreases at higher social support levels.

Aforementioned scaling issues can be addressed by analyzing item-level data through the application of an item response theory (IRT) measurement model within variance decomposition models (i.e., in a unified model; Van den Berg et al. 2007). In short, IRT models can be conceptualized as a latent factor model for ordinal data (i.e., item responses) in which a normally distributed, continuous latent variable (θ) is assumed to reflect the underlying trait of interest. The probability of endorsing a certain item response category is a function of the latent trait (θ) of the individual and the characteristics (parameters) of an item. In the simplest of IRT models for binary data, the probability of endorsing an item (e.g., achieving a score of “agree” = 1 vs. “disagree” = 0) is a function of the difference between an individual’s trait standing (θ) and item “difficulty” parameter *β*_*i*_. IRT models also exist for ordered categorical responses resulting from responses to Likert scales, each with their own distinct set of item parameters (e.g., Samejima 1969; Muraki 1992).

Using IRT models in variance decomposition models has the advantage that it can account for heterogeneous measurement error at different levels of θ (Van den Berg et al. 2007). This is because in IRT models, reliability is not a fixed number for a given instrument, but dependent on the trait level (θ) and the parameters of the instruments’ items. Take a 10-item well-being questionnaire with strongly formulated items (e.g., “I am always happy” as opposed to “I am usually a happy person”) as an example; in this case, many individuals from the general population will receive low scores on the questionnaire, even though of course well-being differences between them exist. The questionnaire will thus not discriminate well and provide little information at lower trait levels (i.e., low reliability/more measurement error), and discriminate better between higher or lower scoring individuals at higher trait levels (i.e., high reliability/less measurement error).

Using simulations Molenaar and Dolan (2014) and Schwabe and Van den Berg (2014) showed that spurious (unmeasured) GxE effects due to skewed sum scores were removed and true parameter values recovered when item-level IRT models were implemented. Extending to the measured GxE case, Schwabe et al. 2017b showed that the shared environmental influences (C) on mathematical ability varied by family socio-economic status. Such methods have, however, not been applied to GxE effects with respect to well-being and social support, while this is especially relevant given the aforementioned skewness of well-being measures.

## The present study

In this study, the influence of using skewed sum scores on gene-environment interactions between well-being and social support is investigated. We focus on satisfaction with life (SWL) and subjective happiness (SH) as measures of well-being as both are often used as general well-being assessments and accompanied by their own reliable, well validated instrument. Models are fitted based on sum scores and on item-level data by incorporating an item response theory measurement model, and results across these two methods are compared to assess the biases introduced by the former. First, unmeasured GxE analyses are conducted for all three variables (life satisfaction, happiness, and social support) to investigate the influence of scaling issues on spurious (univariate) GxE effects. Next, biometric GxE models with social support as the moderator are fitted to test whether estimated GxE effects change when item-level data is analyzed compared to sum scores.

## Method

### Sample

The data for this study come from voluntary participants of the Netherlands Twin Register (NTR), in which twins and their family members periodically (~ every 2 years) fill out questionnaires on their health, lifestyle, well-being, personality, and various other life domains (Ligthart et al. 2019). For this study, we used the 6th wave (collected between 2002 and 2004) and 8th wave of data collection (collected between 2008 and 2010). These waves were selected based on the availability of relevant well-being and social support variables. In principle, we used data from the 8th wave; however, if data were missing, the data from the 6th wave were used for each single twin if present. For the phenotypic IRT analyses (described below) of the SHS, SWLS, and social support scale, we used all available data from twins with complete data for all of the scales’ items (*N*_SHS_ = 5,324, *N*_SWLS_ = 11,216, *N*_*social support*_ = 7763).^2^ For our biometric models, we allowed for one missing item on each scale per twin. For our (univariate) unmeasured GxE analyses this resulted in a total of 11,305 twins for the SWLS (5349 MZ twins, 5956 DZ twins, 4011 complete twin pairs (2134 MZ, 1877 DZ) and 3283 single twins (1081 MZ, 2202 DZ), from 7294 families). For the SHS, data were available for a total of 5463 individual twins (2673 MZ twins, 2790 DZ twins, 1817 complete twin pairs (997 MZ, 820 DZ) and 1829 single twins (679 MZ, 1150 DZ), from 3646 families). For social support, our sample consisted of 7897 individual twins (3919 MZ twins, 3978 DZ twins, 2579 complete twin pairs (1449 MZ, 1130 DZ) and 2739 single twins (1021 MZ, 1718 DZ), from 5318 families).

In our analysis method, missing values are imputed during the estimation process (described below). To not overly rely on imputed values but instead on observed data points, in our measured GxE analyses (i.e., models including social support as the moderator), we limited our analyses to twin pairs with sufficient data available. More specifically, analyses were limited to twin pairs with either a within-twin cross-trait or cross-twin cross-trait correlation present (e.g., SWL_twin 1_ – Social support_twin1_ or SWL_twin 1_ – Social support_twin2_). This ensured that our estimations were mostly data-based. The bivariate SWLS-social support sample consisted of 8528 twins (4221 MZ twins, 4307 DZ twins, 3235 complete twin pairs (1761 MZ, 1474 DZ) and 2058 (699 MZ, 1359 DZ) single twins from 5293 families). The bivariate SHS-social support sample consisted of 3610 twins (1921 MZ twins, 1689 DZ twins, 1606 complete twin pairs (894 MZ, 712 DZ) and 398 (133 MZ, 265 DZ) single twins from 2004 families). Table 1 reports the sample sizes and descriptive statistics of the different sub-samples used in the different analyses.

**Table 1:** Sample characteristics

	Dataset				
	Univariate SH (*N *= 5463)	Univariate SWL (*N *= 11,305)	Univariate social support (*N *= 7897)	Bivariate SH—Social support (*N *= 3610)	Bivariate SWL—Social support (*N *= 8528)
*N *MZM	764 (14%)	1547 (14%)	1069 (14%)	496 (14%)	1160 (14%)
*N *DZM	453 (8%)	994 (9%)	657 (8%)	258 (7%)	708 (8%)
*N *MZF	1909 (35%)	3802 (34%)	2850 (36%)	1425 (39%)	3061 (36%)
*N *DZF	1033 (19%)	2164 (19%)	1499 (19%)	697 (19%)	1632 (19%)
*N *DOS	1304 (24%)	2798 (25%)	1822 (23%)	734 (20%)	1967 (23%)
% female total	64%	64%	65%	68%	65%
Mean age total (*SD*) [range]	33.1 (13.0) [17–85]	30.8 (13.9) [17–97]	32.5 (14.1) [17–90]	37.4 (11.7) [17–80]	32.5 (14.1) [17–90]

*SHS* subjective happiness, *SWL *satisfaction with life

### Measures

**Fig. 1:**
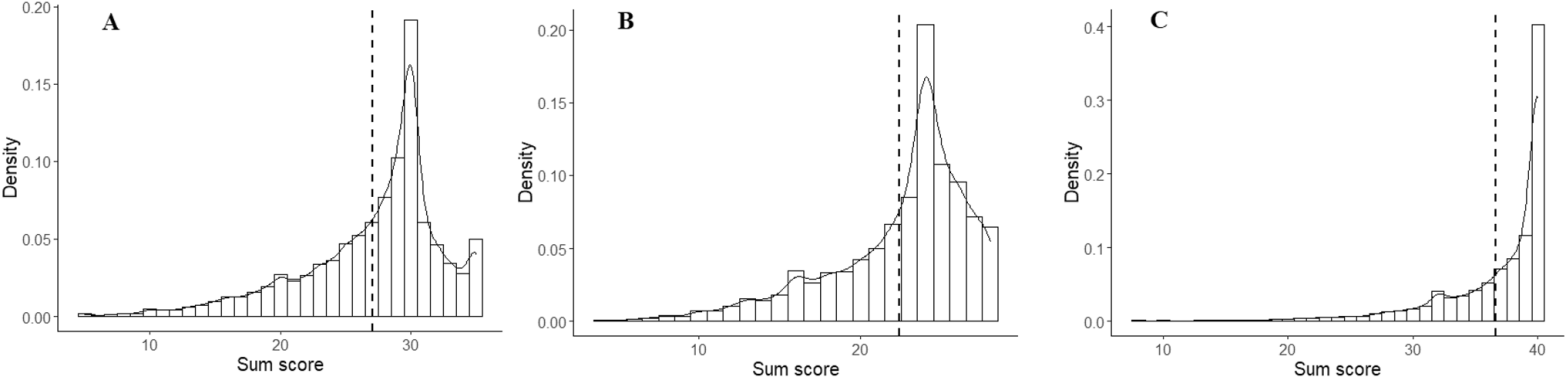
Histogram and density plots of the Satisfaction With Life Scale (**A**), Subjective Happiness Scale (**B**), and social support (**C**) scores. Dashed line indicates the mean

The Satisfaction with Life scale (SWLS; Diener et al. 1985) was used to measure life satisfaction, which includes 5 items rated on a 7-point Likert scale. Life satisfaction captures the cognitive component of Diener’s three-part conceptualization of well-being, the remaining components being presence of positive affect and absence of negative affect (Diener 1984). All SWL items are positively formulated (e.g., indicative) with higher scores representing higher well-being levels. An example item for the SWLS is “*I am satisfied with my life*”. The SWLS sum score distribution (Fig. 1A) was highly skewed to the left (skewness and kurtosis statistics were − 1.14 and 4.31, respectively). More specifically, 5.01% of the sample received the maximum scale score, while 41.08% of the sample rated every item with response option 6 (out of 7) or higher. Reliability of the scale was high (Cronbach’s alpha = 0.88).

The 4-item Subjective Happiness Scale (Lyubomirsky and Lepper 1999), also using a 7-point Likert scale, was used to measure (subjective) happiness. Subjective happiness reflects one’s global, subjective assessment of whether one is a happy or an unhappy person. It is proposed to reflect the frequency of experiencing positive emotions, moods, and affect over time (Lyubomirsky et al. 2005; Diener et al. 2009). Two of the SHS items were non-indicative and were therefore reverse-coded before analyses. An example item for the SHS is “*In general, I consider myself a happy person*”. For the SHS, the skewness and kurtosis statistics were − 1.21 and 4.26 respectively, also indicating a highly left-skewed distribution (Fig. 1B). In this case, 6.46% of the sample received the maximum scale score, while 54.38% rated every item with response option 6 or higher. The reliability was slightly lower for the SHS compared to the SWLS (Cronbach’s alpha = 0.85), but the SHS included one item less.

Social support was measured using the Duke-UNC Functional Social Support Questionnaire (FSSQ; Broadhead et al. 1988) which consists of 8 items to rate the amount of support received on a 5-point Likert scale (1: much less than I would like, 2: less than I would like, 3: somewhat less than I would like, 4: almost as much as I would like, 5: as much as I would like). An example item states “*I get valuable advice about important things in life*.”. The skewness and kurtosis statistic of the social support sum score were − 2.19 and 8.96, respectively, showing an extreme ceiling effect (Fig. 1C) with the modal response being the highest possible score (received by 40.33% of the sample). No less than 83.61% of the sample choose response option 4 out of 5 (“almost as much as I would like”) or higher on all items. Reliability of this scale was good based on the alpha coefficient (0.89).

### Analyses

For all analyses, the statistical programming language R (R Core Team 2020) was used.

#### Phenotypic IRT analyses

To investigate reliability across the trait continuum, i.e., to get an estimate of the level of heterogeneous measurement error, phenotypic IRT analyses were conducted with the *mirt* package (Chalmers 2012). To this end, test information plots were investigated. 3 Although in IRT models the reliability of the scale varies across different trait levels, we also report a single, average reliability indicator provided by the *empirical reliability* statistic (Du Toit 2003). The most commonly used models for Likert scale data are the Graded Response Model (GRM; Samejima 1969) and the Generalized Partial Credit Model (GPCM: Muraki 1992). We used both models to estimate the item parameters and subsequently compared their model fit values: because the GRM showed superior fit (Table S1), we used this model throughout all our analyses.

#### Unified IRT-variance decomposition models

Because of the complexity of our models, Bayesian statistical modelling based on Markov Chain Monte Carlo (MCMC) estimation is used (Van den Berg et al. 2007). In the Bayesian approach, statistical inference is based on the posterior density of all model parameters which is proportional to the product of a prior probability and the likelihood function of the data (see e.g., Box and Tiao 2011, for more information). Following previous studies (e.g., Schwabe and Van den Berg 2014), we used Gibbs sampling, an MCMC algorithm, to study the posterior densities of model parameters. The freely available MCMC software package *JAGS* (Plummer 2003) in combination with the R packages *jagsUI* (Kellner 2019) and *rjags* (Plummer 2019) were used to estimate our models.

In all the analyses described below, we decided not to model shared environmental variance (C) or non-additive genetic variance (D). Previous meta-analyses and reviews have shown little to no effect of C on (adult) well-being (Bartels 2015) and mixed evidence for social support (dependent on the type of support; Coventry et al. 2021). With respect to non-additive genetic variance, evidence is mixed at best for well-being (Stubbe et al. 2005; Bartels and Boomsma 2009; Hahn et al. 2013) and absent for social support (Coventry et al. 2021). A further complicating factor for estimating non-additive genetic effects is the large sample sizes needed to detect them, and our SHS sample (see Table 1) was relatively small. Finally, our twin correlations (Table S4) indicated little to no evidence for the presence of shared environmental variance and non-additive genetic effects. Taken together, we decided to only estimate AE models.

**Fig. 2:**
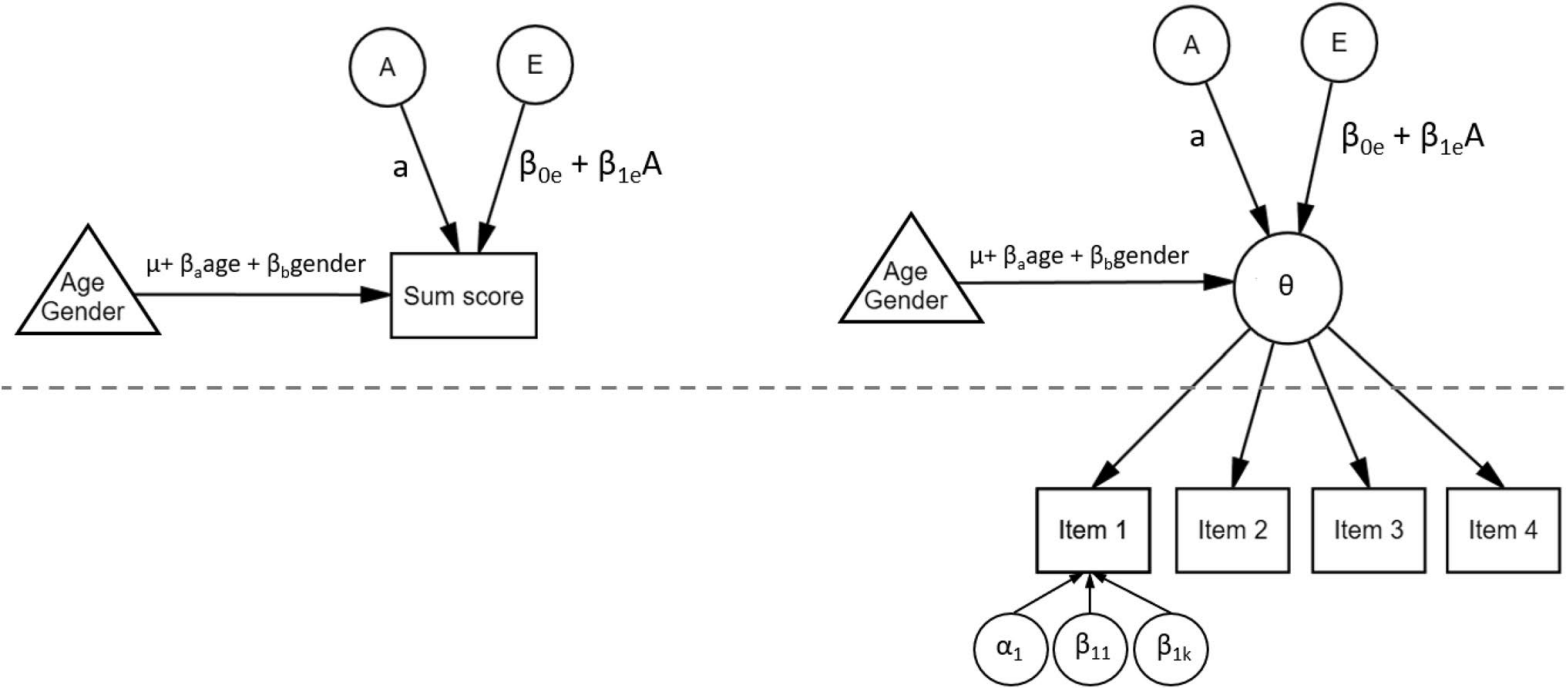
Unmeasured GxE model for a single twin, for sum scores (left) and item-level data (right), where θ represents the latent phenotype. Above the dashed line represents the twin model, below the dashed line the IRT model. For clarity, only the item parameters of Item 1 are given (with discrimination/slope parameter α_1_ and threshold parameters β_1_ through β_*k*_ with *k* being equal to the number of response options *M* − 1. The models without GxE are estimated by replacing β_0e_ + β_1e_A with *e*

Following common practice, age (standardized) and gender were included as covariates in all models described below by regressing them out from the means of the phenotypes within the genetic models (see for example, Fig. 2). All analysis scripts are openly available (see https://osf.io/6wt8j/).

#### Unmeasured (univariate) GxE models

For detecting unmeasured GxE interaction, we used the one-step model developed by Schwabe and Van Den Berg (2014). In our models, a latent phenotype is estimated based on item-level data using an item-response theory model (Fig. 2, right panel). In the AE version of this model, part of the variance in the latent phenotype due to E (σ^2^_E_) varies systematically across different values of A to model AxE by partitioning the E variance component into an intercept (the environmental variance when A = 0) and a part that varies linearly with A. This results in a variance of σ^2^_E_ that is different for each individual twin pair *i* for MZ twins (σ^2^_E*i*_ = exp(β_0_ + β_1_A_*i*_)) and each individual twin *j* for DZ twins (σ^2^_E*ij*_ = exp(β_0_ + β_1_A_*ij*_)). If β_1_ is not significantly different from zero there is no GxE effect present, and the sign of β_1_ indicates whether E is larger at lower (negative β_1_) or higher (positive β_1_) genotypic values. The exponential function is used pragmatically to avoid negative variances (e.g., SanCristobal-Gaudy et al. 1998; Hessen and Dolan 2009).

### Measured GxE models

**Fig. 3:**
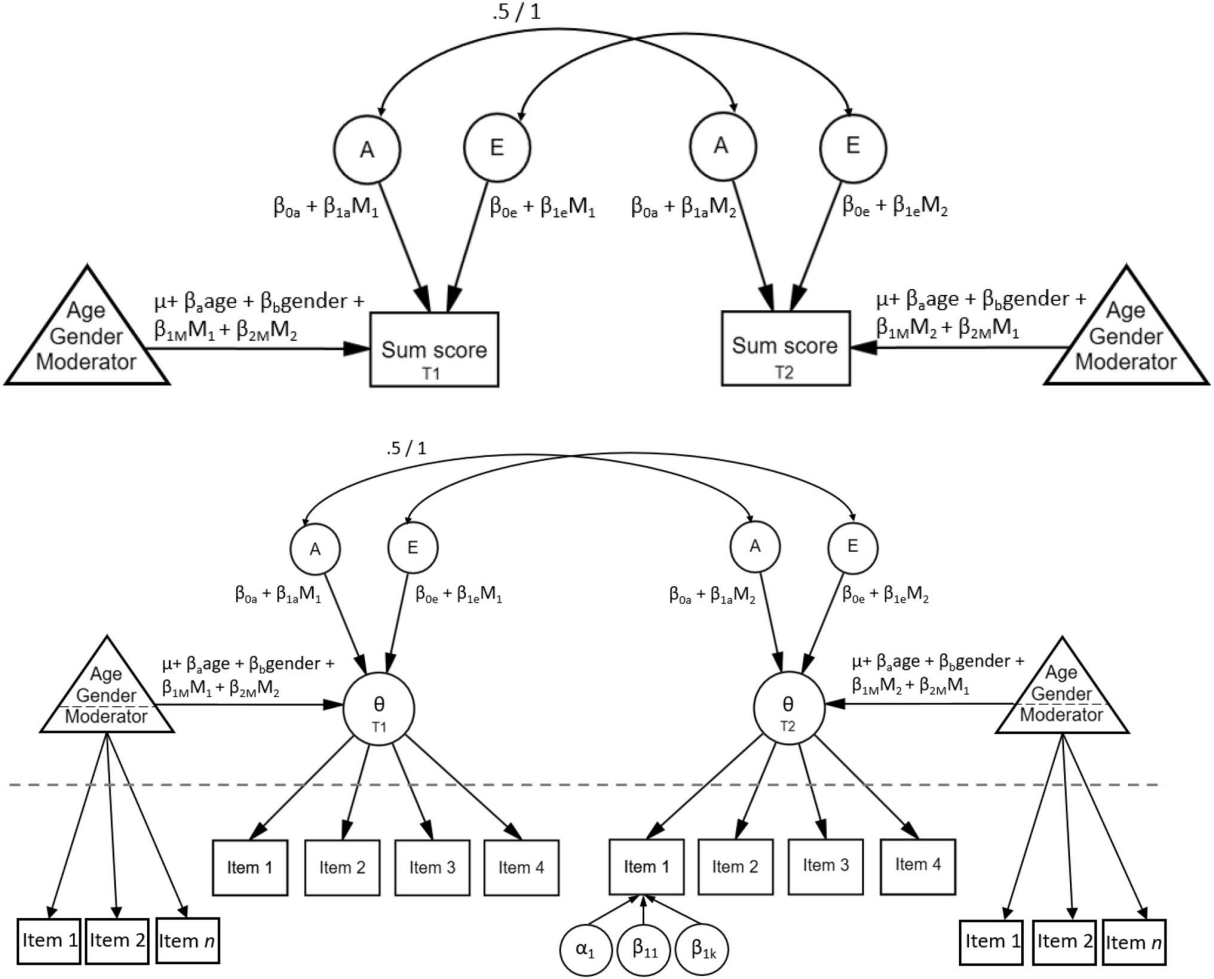
Measured GxE model with social support as moderator for both twins, for sum scores (top) and item-level data (bottom), where θ represents the latent phenotype. Above the dashed line represents the twin model, below the dashed line the IRT model (bottom panel). See Fig. 2 for explanation of the item parameters

Although different variations of biometric GxE models exist for moderators that can differ between twins, we used the model proposed by Van der Sluis et al. (2012) for empirical (e.g., relatively low correlation between social support and happiness) and power considerations. In this model, the main effect of the moderator (M) on the focal trait is incorporated by including its main effect in the means part of the model. More specifically, values on M for both MZ and DZ twins are regressed out of each other’s trait scores (Fig. 3). Moderation of the variance components is included by adding linear effects of M on the paths. Since any covariance between M and the trait is regressed out, GxE effects are tested after gene-environment correlation is accounted for. In other words, the effects of M on the residual variance components are modeled.^4^

We use the model parametrization by Schwabe et al. (2017), who showed that the original moderation model proposed by Purcell (2002) is not uniquely identified, modeling moderation on the log-transformed variances (e.g., σ^2^_E_ = exp(*β*_0*e*_ + *β*_1*e*_M_*ij*_)). We adapted the model by Schwabe et al. 2017b, who included a binary-coded moderator that did not differ between twins, to include an item-level IRT model for a moderator (social support) that did differ between twins. This was achieved by estimating correlated latent phenotypes for both twins based on the responses on the items of the social support scale with an IRT model (Fig. 3, bottom panel). In all unified IRT variance decomposition models, the population mean (µ) was set to 0 and the first item discrimination parameter to 1 for identification, allowing for free estimation of the latent variance components. Further model details can be found in Schwabe et al. 2017a and in our analysis scripts.

### Sum score analyses

Because we were interested in the differences in parameter estimates when item-level data were analyzed instead of sum scores, in addition to the models described above, we estimated all models using sum scores. In these models, sum scores were calculated from the twin data by summing the item scores after which these were standardized to have a mean of zero and variance of one. Standardization was done to make results of both approaches comparable with respect to their prior distributions. Sum scores were then analyzed with the same *JAGS* script as for the item-level data but without the IRT part; instead of estimating θ_*ij*_ with an IRT model, sum scores were used as directly observed estimates of the phenotypes (ignoring measurement error). The unmeasured GxE model for sum scores is shown in Fig. 2 (left panel) and the measured GxE model in Fig. 3 (top panel).

### Estimation procedure

In the Bayesian estimation framework, prior distributions for the unknown model parameters have to be made explicit. For our prior distributions, we followed previous studies and used prior distributions that are relatively uninformative (Schwabe and Van den Berg 2014; Schwabe et al. 2017b). Prior probability densities applied in our study can be found in the analysis scripts.

The characterization of the posterior distribution was based on a total of 25,000 iterations from two different Markov chains (i.e., in total 50,000 iterations), after a burn-in phase of 30,000 iterations for each separate Markov chain.^5^ Posterior point estimates of the model parameters were calculated by taking their mean over all iterations, and their standard deviations are also reported. The 95% highest posterior density (HPD; see for example, Box and Tiao 2011) interval, which can be interpreted as the Bayesian equivalent of a confidence interval (CI) was also calculated for all relevant parameters. Analog to the CI, parameters can be regarded as significant when its HPD interval does not contain zero. This does not hold for the variance components, as these by definition have a lower-bound of zero. To assess and compare model fit, the deviance information criterion (Spiegelhalter et al. 2002) was used. The DIC can be regarded as the hierarchical modeling generalization of the Akaike information criterion (AIC), and can be used for Bayesian model selection with lower values indicating more parsimonious models.

In all our models, missing item data was assumed to be missing at random. A convenient feature of MCMC estimation methods is that it can account for missing data. At each iteration, the latent genetic and environmental values, but also observed covariates – including missing values – of individual twins are imputed conditional on the data and the model parameters (Eaves and Erkanli 2003). The only requirement for handling missing observed data this way is that prior distributions are used for the covariate values for which observations are missing. To account for missing age values, we placed relatively non-informative priors on the standardized age covariate (N(0,10)) in all models. The same prior was used for the social support variable in the sum score measured moderator model.

## Results

### Heterogeneous measurement error

The test information plot (Fig. S3) for the SWLS showed that information (i.e., reliability) was highest at lower trait levels (highest around − 1.5), lower around average levels, and higher again around above average (trait = 1) levels. Overall, the IRT-based reliability was 0.89. For the SHS, we also found that test information was highest at lower trait levels (highest between − 2 and − 1), with a decrease around average levels, and with a slight increase around above average (θ = 1) levels (Fig. S6). The IRT-based reliability was 0.86, but note that the SHS contains one item less than the SWLS. Overall, the SWLS and SHS showed a similar pattern of reliability across the trait continuum, with (a) the peaks and valleys being more pronounced for the SWLS and (b) higher information levels in general for the SWLS. Finally, for social support, information was only provided at lower trait levels (between − 3 and 0), while from average levels onward (> 0) the scale provided virtually no information (Fig. S9). This lack of information at some trait levels led to a relatively low average reliability as indicated by an IRT-based reliability of 0.74.

### Unmeasured (univariate) GxE

Table 2 shows the estimated parameters from the univariate unmeasured GxE models, using the sum score and item-level approach. The results were in line with what is to be expected under spurious GxE due to ceiling effects. That is, using the sum score approach, there appeared to be strong negative GxE interaction effects for both well-being scales (β_1*SWL*_ = −1.74 [−1.83−1.66] and β_1*SHS*_ = −1.98 [−2.10−1.85]), implying reduced environmental variance (i.e., less individual differences) at higher genotypic values. The larger (spurious) GxE interaction effect (β_1_) for the SHS compared to the SWLS is to be expected based on differences in the scales’ skewness. The spurious negative interaction effect was largest (β_1_ = −4.45 [−4.62−4.29]) for the most heavily skewed social support variable.

**Table 2:** Posterior point estimates, standard deviations, 95% HPD intervals, and DIC values for un^i^variate (unmeasured) GxE models

	SWL				SHS				Social support			
	Sum		Item		Sum		Item		Sum		Item	
*Parameter*	PPE (SD)	95% HPD	PPE (SD)	95% HPD	PPE (SD)	95% HPD	PPE (SD)	95% HPD	PPE (SD)	95% HPD	PPE (SD)	95% HPD
β_sex_	− 0.01 (0.01)	− 0.02–0.01	− 0.09 (0.03)	− 0.15 - − 0.03	− 0.02 (0.01)	− 0.04–0.00	− 0.15 (0.04)	− 0.24 − 0.07	0.01 (0.01)	− 0.00–0.02	0.06 (0.03)	− 0.01–0.12
β_age_	− 0.01 (0.01)	− 0.03–0.00	− 0.10 (0.03)	− 0.16 - − 0.04	− 0.03 (0.01)	− 0.05 - − 0.01	− 0.11 (0.05)	− 0.19 - − 0.02	− 0.00 (0.01)	− 0.02–0.01	− 0.20 (0.03)	− 0.26 - − 0.13
σ^2^_A_	0.34 (0.01)	0.32–0.36	3.33 (0.20)	2.95–3.72	0.32 (0.01)	0.30–0.35	3.37 (0.31)	2.78–4.02	0.20 (0.01)	0.19–0.22	3.07 (0.25)	2.59–3.57
exp(β_0_)	0.40 (0.01)	0.38–0.42	4.71 (0.21)	4.30–5.14	0.37 (0.01)	0.35–0.40	4.49 (0.34)	3.85–5.16	0.23 (0.01)	0.22–0.24	4.28 (0.27)	3.76–4.81
β_1_	−1.74 (0.04)	−1.83 - −1.66	0.05 (0.02)	0.01–0.08	− 1.98 (0.06)	− 2.10 - −1.85	− 0.06 (0.03)	− 0.12 - − 0.01	− 4.45 (0.08)	− 4.62 - − 4.29	0.54 (0.03)	0.48–0.59
*h* 2	0.46 (0.01)	0.43–0.48	0.41 (0.02)	0.38–0.45	0.47 (0.02)	0.43–0.50	0.43 (0.03)	0.37–0.48	0.47 (0.01)	0.44–0.49	0.42 (0.02)	0.37–0.47
DIC	94447.86		176565.7		46358.61		74816.19		62642.1		113624.1	

*SWL*   satisfaction with life, *SHS*  happiness, *h*^2^  heritability, *PPE *  point posterior estimate, *HPD*   highest posterior density, *SD*  standard deviation, *DIC  * the deviance information criterion

**Fig. 4:**
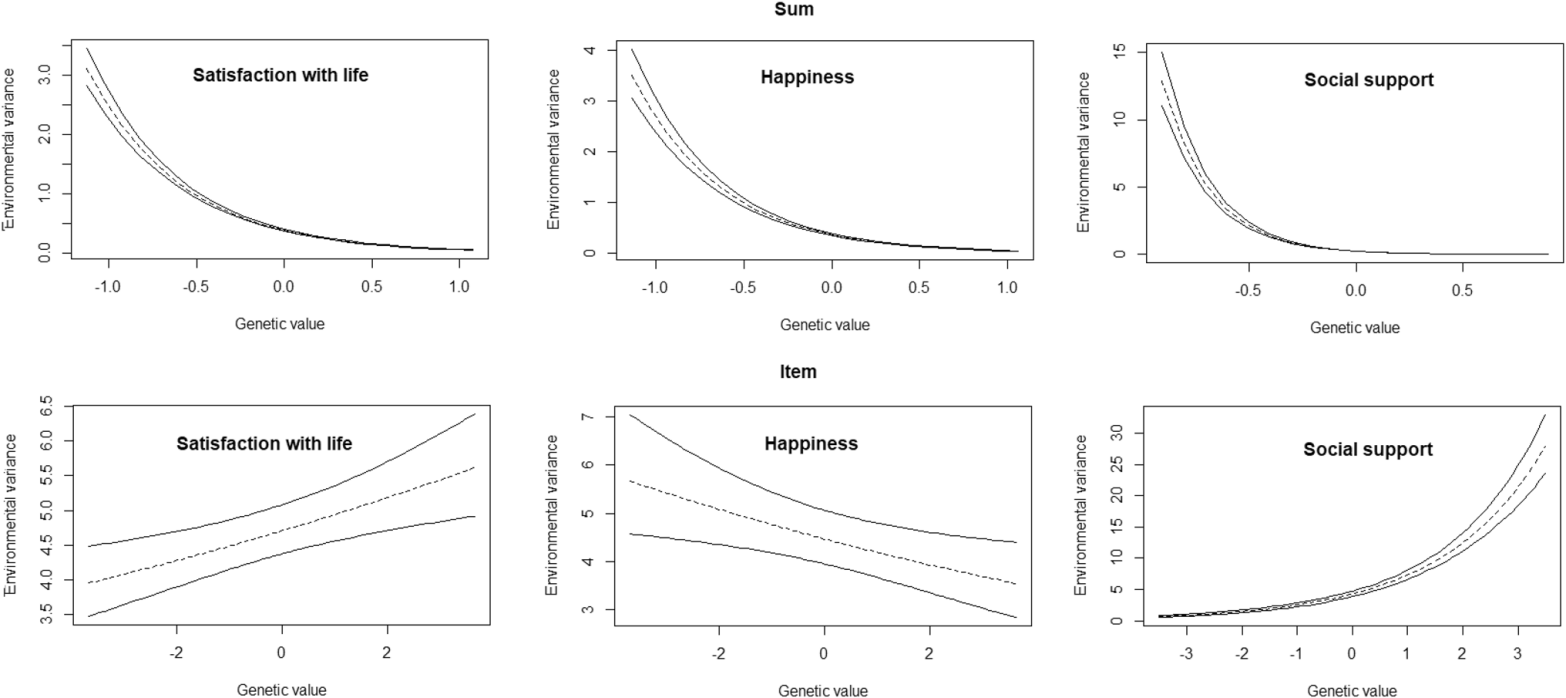
95% credibility regions of the AxE interaction effects across the range of estimated genotypic values based on sum scores (top) and item-level data (bottom) for satisfaction with life, happiness, and social support

Using the item-level approach, the negative interaction effects disappeared for SWL (β_1_ = 0.05; [0.01–0.08]) and SH (β_1_ = − 0.06; [−0.12−0.01]). Although the HPD intervals of β_1_ did not contain zero for both traits, the univariate models without GxE (effectively setting β_1_ = 0) had lower DIC values (Table S3), indicating superior model fit. In contrast, with sum scores, DIC values were lower for the models including the GxE effects (β_1_). Interestingly, for social support, a positive and significant GxE effect was found in the item-level model (β_1_ = 0.54; [0.48−0.59]), indicating that individuals with higher genotypic values show more variance due to unique-environmental influences (see Fig. 4 for a graphical representation).

### Measured GxE with social support as moderator

Table 3 shows the estimated parameters from the measured moderator model (left panel; sum scores, right panel; item-level).

**Fig. 5:**
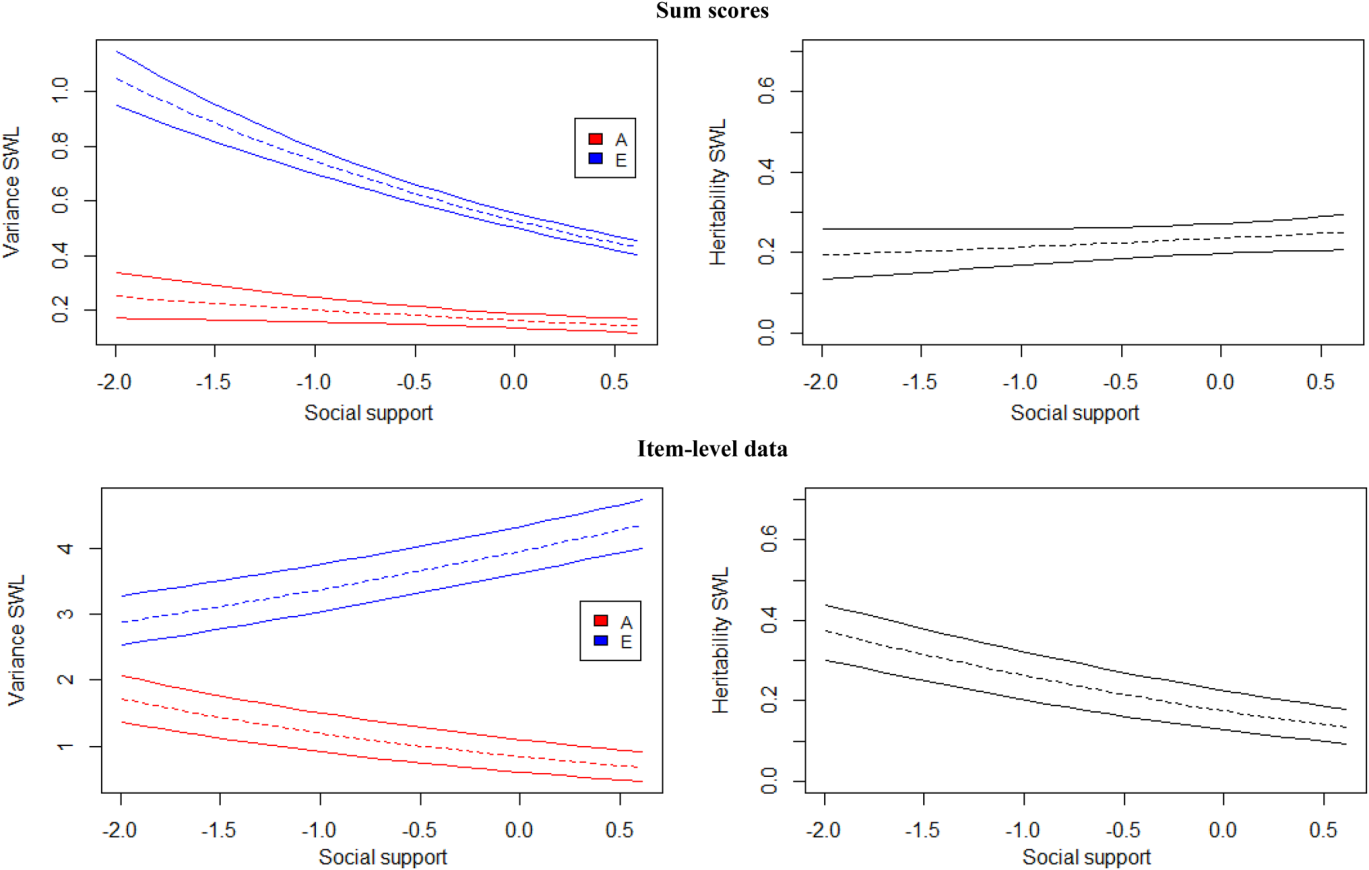
95% credibility regions of the GxE interaction effects across the range of social support based on sum scores (top) and item-level data (bottom) for satisfaction with life (SWL). Standardized values in right panel

**Fig. 6:**
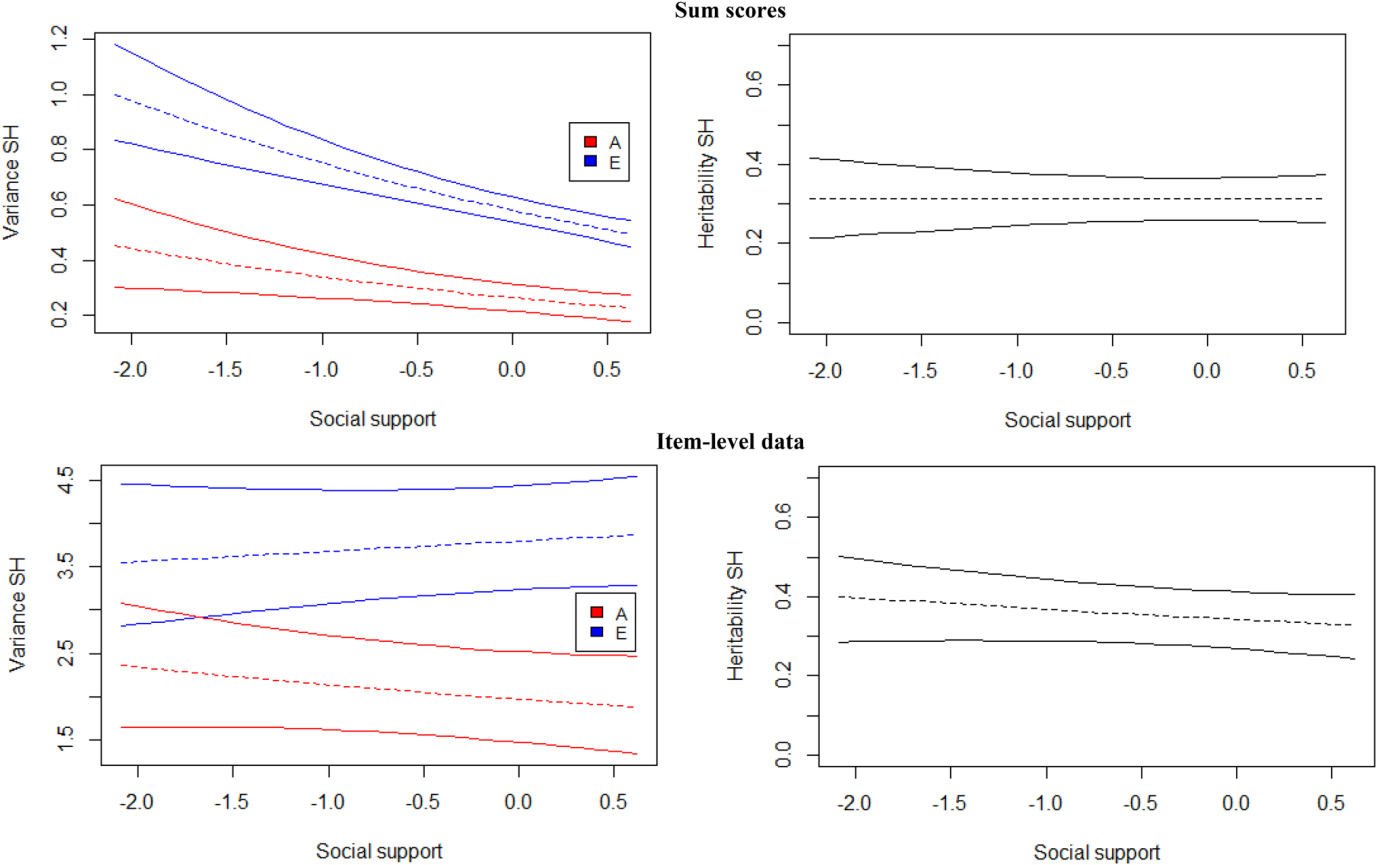
95% credibility regions of the GxE interaction effects across the range of social support based on sum scores (top) and item-level data (bottom) for subjective happiness (SH). Standardized values in right panel

When sum scores are analyzed, for both life satisfaction (β_1a_ = − 0.21; [−0.37−0.03]) and happiness (β_1a_ = − 0.25; [−0.43−0.06]), the amount of additive genetic variance decreases with increasing social support (Figs. 5 and 6, top panels). A negative effect is found for the amount of environmental variance, i.e., E decreases with higher social support levels (β_1e_ = − 0.34; [−0.39−0.29] for SWL, β_1e_ = − 0.26; [−0.35−0.17] for SH). Together, these moderation patterns do not lead to noticeably different (standardized) heritability estimates across different levels of social support for both happiness and life satisfaction (Figs. 5 and 6, top right panels).

**Table 3:** Posterior point estimates, standard deviations, 95% HPD intervals, and DIC values for measured GxE model parameters

	SWL				SHS			
	Sum		Item		Sum		Item	
Paramete*r*	PPE (SD)	95% HPD	PPE (SD)	95% HPD	PPE (SD)	95% HPD	PPE (SD)	95% HPD
β_sex_	− 0.03 (0.01)	− 0.05 -− 0.01	− 0.15 (0.03)	− 0.21 - − 0.09	− 0.06 (0.02)	− 0.10 - − 0.03	− 0.24 (0.05)	− 0.34 − 0.13
β_age_	− 0.01 (0.01)	− 0.03–0.01	0.00 (0.03)	− 0.06–0.06	− 0.01 (0.02)	− 0.05–0.02	0.02 (0.05)	− 0.08–0.12
β_1MZ_	0.51 (0.02)	0.47–0.54	0.65 (0.03)	0.59–0.71	0.33 (0.03)	0.27–0.38	0.39 (0.04)	0.31–0.47
β_2MZ_	0.11 (0.02)	0.07–0.14	0.11 (0.02)	0.06–0.15	0.11 (0.03)	0.05–0.16	0.10 (0.04)	0.03–0.17
β_1DZ_	0.52 (0.02)	0.49–0.55	0.67 (0.03)	0.62–0.73	0.34 (0.03)	0.28–0.40	0.41 (.04)	0.33–0.49
β_2DZ_	0.04 (0.02)	0.00–0.08	0.03 (0.03)	− 0.02–0.03	0.06 (0.03)	− 0.00–0.12	0.08 (0.04)	0.01–0.16
exp(β_0a_)	0.16 (0.02)	0.13–0.19	0.84 (0.15)	0.55–1.15	0.26 (0.03)	0.21–0.32	1.98 (0.32)	1.35–2.61
β_1a_	− 0.21 (0.09)	− 0.37– −0.03	− 0.36 (0.04)	− 0.45 - − 0.28	− 0.25 (0.09)	− 0.43 - − 0.06	− 0.08 (0.08)	− 0.23–0.09
exp(β_0e_)	0.53 (0.02)	0.50 - 0.56	3.96 (0.22)	3.54–4.38	0.58 (0.03)	0.53 - 0.64	3.81 (0.37)	3.11–4.53
β_1e_	− 0.34 (0.03)	− 0.39 - − 0.29	0.16 (0.02)	0.12–0.20	− 0.26 (0.05)	− 0.35 - − 0.17	0.03 (0.05)	− 0.06–0.12
*h*^*2*^	0.24 (0.02)	0.19–0.28	0.18 (0.03)	0.12–0.23	0.31 (0.03)	0.25–0.37	0.34 (0.04)	0.25–0.42
DIC	114744.9		207711.1		44049.49		73933.54	

*SWL* satisfaction with life, *SHS* happiness, *PPE* point posterior estimate, *HPD* highest posterior density, *SD* standard deviation, *DIC* the deviance information criterion, *MZ* monozygotic, *DZ* dizygotic, *h*^2^ heritability

The item-level analyses provide very different results for both traits. For SWL, the moderation of the additive genetic variance is still negative and significant (β_1a_ = − 0.36; [−0.45−0.28]), while the moderating effect of the non-shared environmental variance has become positive and significant (β_1e_ = 0.16; [0.12−0.20]). Thus, as social support increases, the genetic variance decreases, while the environmental variance increases, together resulting in lower heritability estimates (Fig. 5, bottom panel). For happiness, however, the moderating effects (β_1a_ = − 0.08; [−0.23−0.09] and β_1e_ = 0.03; [−0.06−0.12]), while in the same direction as for SWL, are not significant as indicated by their HPD’s including zero. Thus, the heritability of happiness does not seem to depend on one’s level of social support (Fig. 6, bottom panel).

### Additional analyses: bivariate variance decomposition models

Apart from GxE effects, it is also informative to show differences in (bivariate) heritability estimates across methods since previous studies have shown that scaling issues can have a large influence on them (Van den Berg et al. 2007). To this end, two bivariate variance decompositions (happiness-social support, and life satisfaction-social support) were fitted based on sum scores and item-level data (Table S4 and S5, Fig. S10). For both well-being traits, the genetic variance was estimated to be ~ 4% higher when item-level data was analyzed, increasing from 35% [31–38] to 39% [35–43] for SWL and 38% [33–43] to 43% [35–49] for SHS. The largest parameter estimate difference between the sum score and item-level model was found for the heritability of social support in the SWL-social support model, increasing from 35% [0.32–0.39] to 44% [0.39–0.48]. As for the bivariate heritability (i.e., part of the covariance due to additive genetic factors), these increased from 48% [0.42–0.54] to 53% [0.47–0.58] for SWL and social support, and from 54% [0.43–0.64] to 61% [0.48–0.74] for SH and social support. Genetic and environmental correlations between social support and SWL slightly increased (from 0.70 [0.65–0.75] to 0.74 [0.69–0.79], and 0.40 [0.37–0.44] to 0.47 [0.43–0.52], respectively), while for SH-social support the genetic correlation increased more strongly (from 0.50 [0.41–0.60] to 0.60 [0.47–0.73]) and the environmental correlation remained relatively stable (from 0.24 [0.18–0.29] to 0.26 [0.17–0.35]).

## Discussion

In the current study, we investigated the bias introduced by the use of sum scores in GxE findings for well-being with social support. By implementing appropriate item-level IRT models, it was found that skewness of the scales had a large influence on the appearance of spurious GxE effects. First, in the univariate unmeasured case, when sum scores were used, strong negative GxE effects occurred (i.e., less environmental variance at higher genotypic values). However, for life satisfaction and happiness, these effects disappeared when item-level data were used. The spurious effect for happiness was stronger than for life satisfaction, which was expected since the sum score distribution of the former was more skewed, and the effect was strongest for social support, which had the most extremely skewed distribution. Interestingly, when adopting the IRT model we found a positive unmeasured GxE effect for social support, meaning that environmental circumstances become more important as the genetic predisposition for social support increases. Intuitively, this makes sense, as the environmental conditions that foster or hinder social support will only be important for those who are genetically inclined to seek out or enjoy social support. More generally, the differences between sum score and IRT analyses indicate that when one is interested in estimating genotype-environment interaction(s), one should take into account the scale properties of phenotype’s measurement instrument. Although this has previously been shown for other phenotypes (e.g., math ability and affect; Molenaar and Dolan 2014; Schwabe et al. 2017a), this study has shown that this is also important for well-being.

Another important finding was that the moderating effects of social support on the genetic and environmental variance in the two well-being scales differed considerably depending on the method of analysis. The expected moderating effects (for life satisfaction, not happiness, see below) were only found when heterogeneous measurement error was taken into account by implementing the IRT model. Using sum scores, environmental effects decreased at higher social support levels: because of the ceiling effect of both the well-being and social support scales and their positive correlation, it is likely that twins at the higher end of one scale appeared to be more similar on the other scale as well. Consequently, at higher levels of the moderator, unique environmental effects on well-being will appear to be smaller, implying a negative ExM effect. The IRT model adequately controls for these spurious associations. As a result, in the item-level models, the estimated unique environmental variance increased at higher social support levels. These results stress the importance of accounting for heterogeneous measurement error when testing for gene by environment interactions in biometric analyses.

In the item-level models, social support’s moderation of genetic and environmental effects were only found for life satisfaction, not for happiness. This finding could be due to statistical power: the sample size for life satisfaction was more than twice as large as the sample size for happiness, limiting our ability to find the effects for happiness even if they were truly there. This seems plausible since the estimated effects were in the same direction as for SWL, but smaller. An alternative, more substantive explanation could be that life satisfaction is fundamentally different from happiness, and that its unique characteristics are more dependent on social support. This explanation is equally likely, given the stronger phenotypic and genetic associations between SWL and social support compared to happiness. Future studies with larger sample sizes are needed to shed light on the likelihood of the two explanations.

Our study replicates previous findings that social support moderates genetic and environmental effects on well-being, apart from the (genetic) correlation between social support and well-being (Nes et al. 2010; Whisman and South 2017). Heritability estimates of life satisfaction were lower at higher social support levels. These results fit with the *diathesis-stress* model for mental health which posits that stressful environments (i.e., when social support is low) trigger genetic predispositions to be expressed more strongly (Monroe and Simons 1991; Rende and Plomin 1992). Or, framed differently, in stable environments lacking environmental stressors (i.e., at high levels of social support), genetic differences are suppressed and behavior is more likely to be less dispositional (lower A) and more situational (higher E).

In line with previous studies, we found that social support was moderately heritable, indicating gene-environment correlation, and that genes influencing social support partly overlap with well-being. Our item-level models indicated that ~ 50% of the covariance between the two well-being measures and social support was due to genetic effects. This estimate is comparable with previous studies based on sum scores (e.g., Wang et al. 2017). In addition, the genetic correlations were high, and somewhat higher (0.74, item-level data) for life satisfaction than for happiness (0.60, item-level data). Environmental correlations were lower but still sizeable, especially for life satisfaction (0.47, compared to 0.26 for SH, both item-level data). Taken together, our results underline the importance of social support for individual differences in well-being (Diener 2012) at the phenotypic and genetic level.

Although not the main focus of our study, we found that implementing item-level IRT models in lieu of sum scores resulted in slightly higher heritability estimates of the traits. Both happiness and life satisfaction showed highly similar increases in heritability (35%–39% for SWL, and 38%–43% for SHS). These values are in line with previous studies (Bartels and Boomsma 2009; Haworth et al. 2017; Konkolÿ Thege et al. 2017; Wang et al. 2017) showing similar although somewhat lower heritability estimates for happiness (0%–44%) compared to life satisfaction (44%–67%). Although these differences are not too dramatic, relatively small differences in variance component estimates may still translate into larger differences in genetic correlation estimates in bivariate models. For example, we found that the estimate of the genetic correlation between happiness and social support increased from 0.50 (sum scores) to 0.60 (item-level). Thus, researchers may still want to adopt item-level IRT models instead of using sum scores in twin models to get the most accurate point estimates of variance components and genetic correlations.

The extent to which this will affect results will depend on a number of factors. For example, Van den Berg et al. 2007 showed that attenuation due to scaling issues is larger for scales with fewer items. In this regard, the relatively small effect of adopting IRT models we found is surprising, given our relatively short scales (4-item SHS, 5-item SWLS, and 8-item social support scale). In fact, seemingly contrary to Van den Berg et al. 2007, the largest effect on heritability estimates was found for the longest scale, social support (35%–44%, in the bivariate model with SWL). However, this longer social support scale also showed much more skewness than the shorter scales and therefore more heterogeneous measurement error. At the same time, happiness and life satisfaction appeared to be measured with relatively little (heterogeneous) measurement error. Consequently, controlling for heterogeneous measurement error with an IRT model had a larger effect on social support than on happiness and life satisfaction. The expected gains of implementing IRT models instead of skewed sum scores will thus partly depend on the combined effect of scale length and skewness. Another factor to take into account is the specific distributions of the sum scores: in our study, many respondents answered with response option 6 (out of 7) to all items on the SHS and SWLS, and with the highest response option on the social support scale. This will have reduced the scales’ (co)variances affecting both their reliability and (twin) correlations. It remains to be seen whether our results generalize to scales with other distributional properties. Finally, the effect of implementing IRT models on parameter estimates will also depend on the ‘true’ correlations between different traits (Van den Berg et al. 2007). Taken together, the joint effect of a scale’s number of items, reliability, skewness, and the true correlations among traits on variance component estimates should be investigated systematically in the future.

When interpreting our results, the representativeness of our sample should be taken into account as it may limit their generalizability. Our samples included ~ 65% females, which is not representative of the Dutch population (~ 50%). Furthermore, this study used data from the Netherlands, a highly industrialized, western European country and the results may therefore not generalize to other, non-Western, more agricultural contexts. This may be particularly relevant for the social support variable, as previous studies have shown that the relation between social support and well-being differs across cultures depending on the extent to which they can be characterized as being individualistic vs. collectivistic (Brannan et al. 2013). The Netherlands is a highly individualistic country (Hofstede 2001), so additional research is needed to test whether our results extend to more collectivistic cultures.

Due to the nature of our phenotypes and sample (i.e., adults), we only estimated AE models, thus not estimating non-shared environmental effects (C). For the heavily skewed social support scale, the twin correlations based on sum scores indicated an ACE model, while the item-level analyses supported an AE model. Although testing the influence of scaling issues on the comparative sizes of the additive (A), non-additive (D), and non-shared environmental variance (C) was beyond the scope of our study, we deem it a useful effort in the future.

## Concluding remarks

The field of behavior genetics is increasingly investigating the interplay between genes and environments. Incorporating gene by environment interactions within twin models can be a powerful way to do so. However, to achieve unbiased results, the appropriate methods need to be used. In the current study, we have highlighted possible consequences of using skewed sum scores by comparing results from sum score models with item-level IRT models, applied to the field of well-being. We showed that spurious unmeasured GxE effects due to skewness can be an issue and that IRT models can adequately correct for such biases. Finally, item-level GxE models uncovered gene-environment interaction effects indicating that heritability of life satisfaction is lower at higher social support levels. Taken together, this study provides a step forward towards improved estimation methods and understanding of gene-environment interplay in the field of well-being.

## Electronic Supplementary Material

Below is the link to the electronic supplementary material.

Supplementary Material 1

Supplementary Material 2

## Data Availability

NTR data are available for legitimate researchers via a data access procedure (see https://tweelingenregister.vu.nl/information_for_researchers/working-with-ntr-data).

## Footnotes

1 For brevity, we refer to “social support” rather than “perceived social support” from here onwards.

2 We also conducted all our psychometric analyses (IRT, reliability, and skewness) on a reduced sample of one randomly selected twin per family to account for non-independence of data due to family relatedness. Estimates were highly comparable and therefore results on the full sample are reported.

3 Item probability plots and item information plots can be found in Figs. S1 and S2 (SWL), S4 and S5 (SH), and S7 and S8 (social support).

4 Rather than first regressing out the moderator from the mean of the focal trait, a full bivariate decomposition model for the trait and the moderator including interactions can also be estimated. We estimated the full bivariate sum score model in *OpenMx* (Neale et al. 2016) and found that the moderating effects of social support on the cross-paths were insignificant for SH (both A and E effects) and SWL (only the effect on E). With no moderation of the cross-paths, this model is the more adequate model (Van der Sluis et al. 2012). Results can be found in the Table S2.

5 These values were based on test runs with multiple chains and computing Gelman and Rubin’s convergence diagnostic (Gelman and Rubin 1992). All test runs with these numbers of iterations resulted in values below the common < 1.1 rule-of-thumb (Gelman et al. 2004).

## Code availability

All analysis scripts are openly available at https://osf.io/6wt8j/.
