# Supplementary material for: Bias in Gene-by-Environment Interaction Effects with Sum Scores; An Application to Well-being Phenotypes
Source: Behav Genet. 2023 Mar 1;53(4):359–73. doi: 10.1007/s10519-023-10137-y (PMC10275801; doi:10.1007/s10519-023-10137-y)
Supplement: Supplementary file 2 — Supplementary Material 2 [file 10519_2023_10137_MOESM2_ESM.docx]

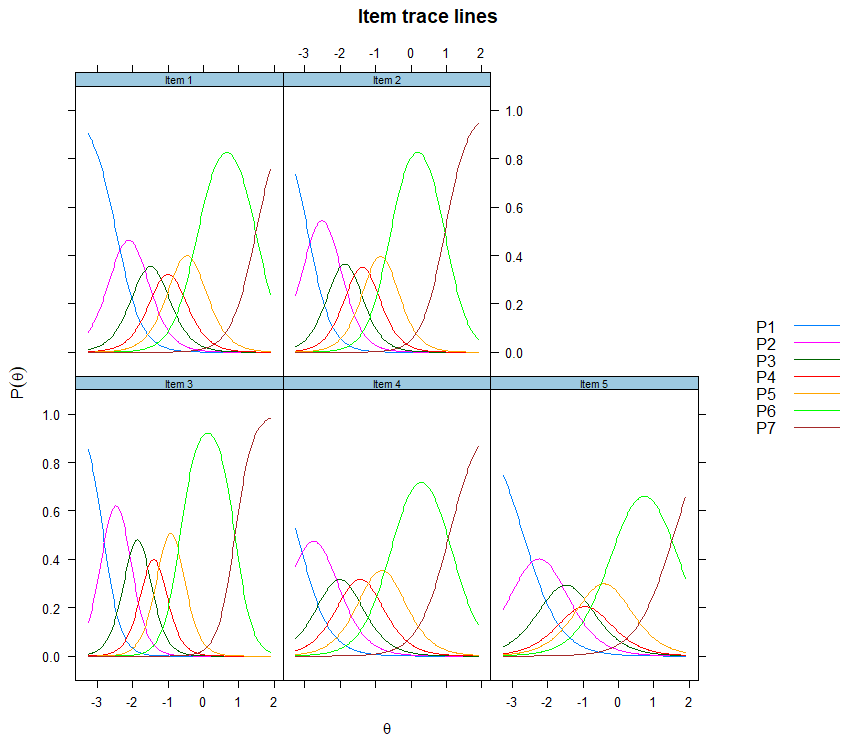


Figure S1. Item probability functions (SWLS).


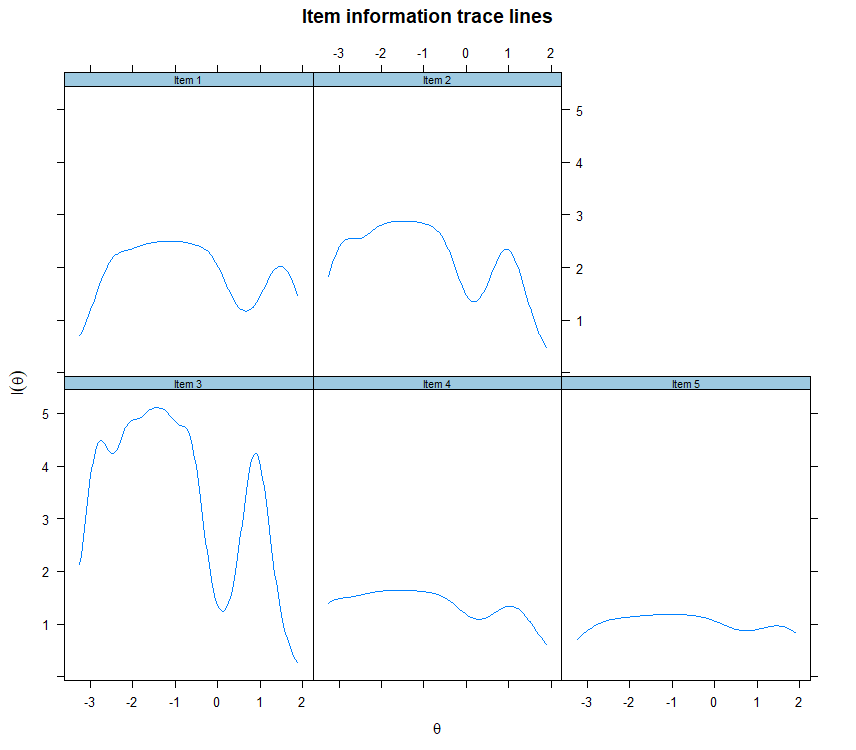


Figure S2. Item information functions (SWLS). Higher information values indicate higher levels of reliability.


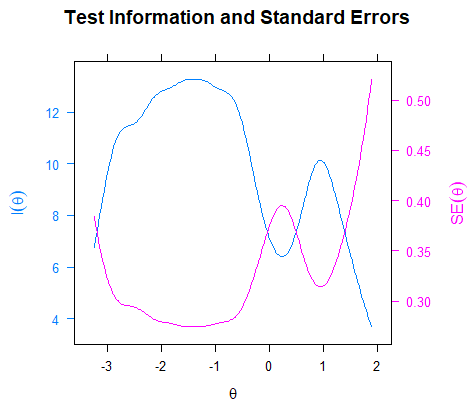


Figure S3. Test information (I: left axis, blue line) and standard errors (SE; right axis, pink line) across the trait continuum θ for SWLS. Higher/lower information/SE values indicate higher reliability.


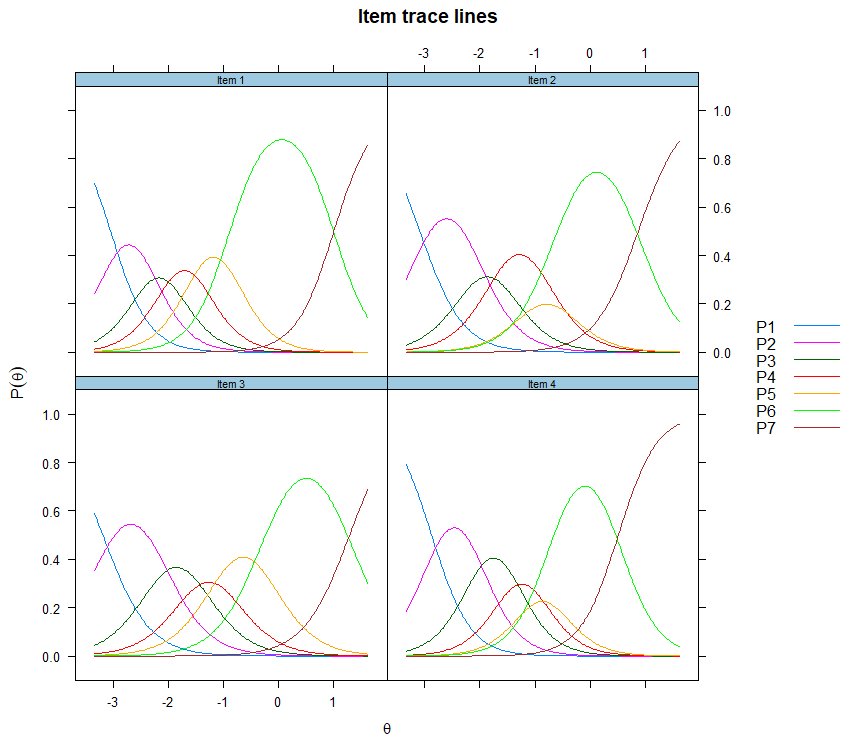


Figure S4. Item probability functions (SHS).


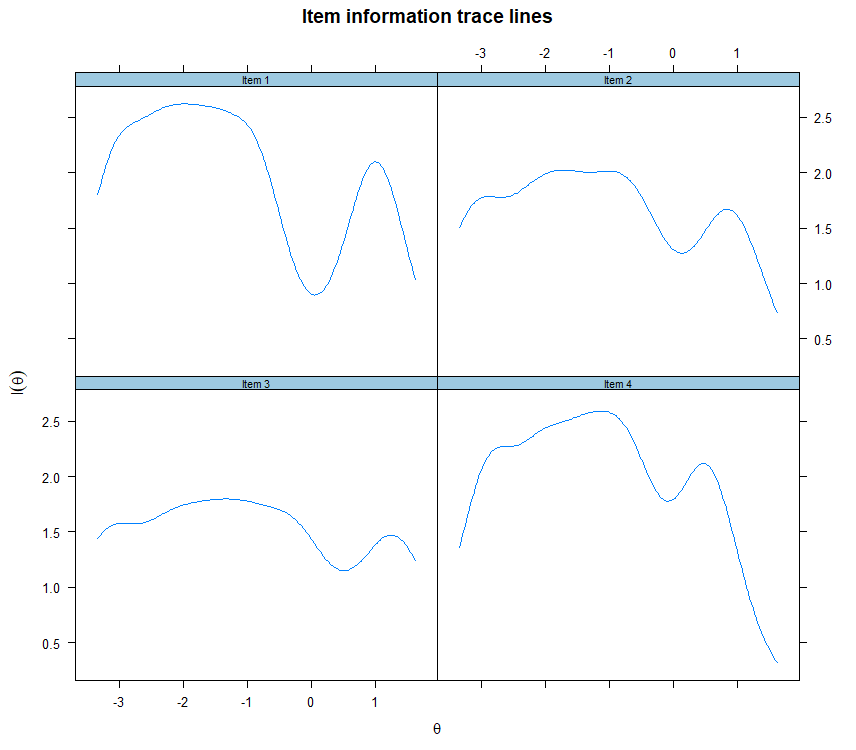


Figure S5. Item information functions (SHS). Higher information values indicate higher levels of reliability.


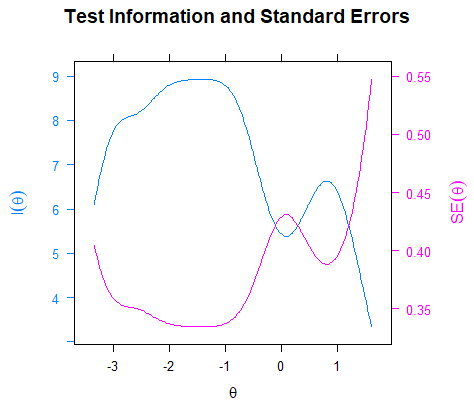


Figure S6. Test information (I: left axis, blue line) and standard errors (SE; right axis, pink line) across the trait continuum θ for SHS. Higher/lower information/SE values indicate higher reliability.


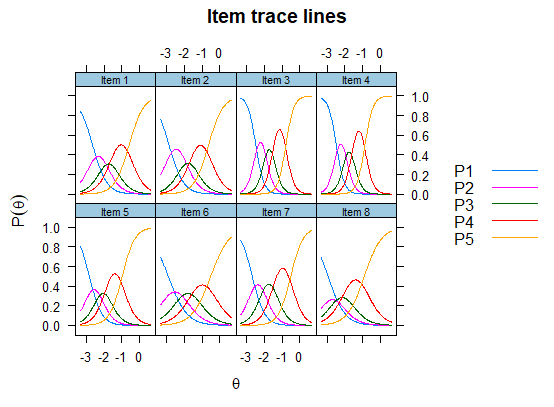


Figure S7. Item probability functions (social support).


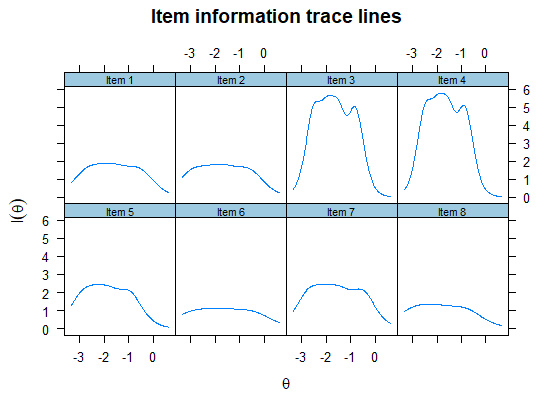


Figure S8. Item information functions (social support). Higher information values indicate higher levels of reliability.


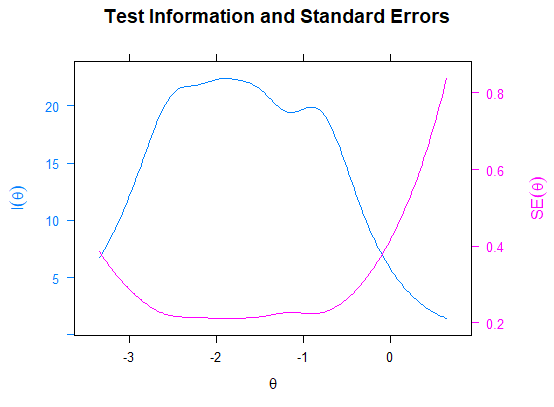


Figure S9. Test information (I: left axis, blue line) and standard errors (SE; right axis, pink line) across the trait continuum θ for social support. Higher/lower information/SE values indicate higher reliability.


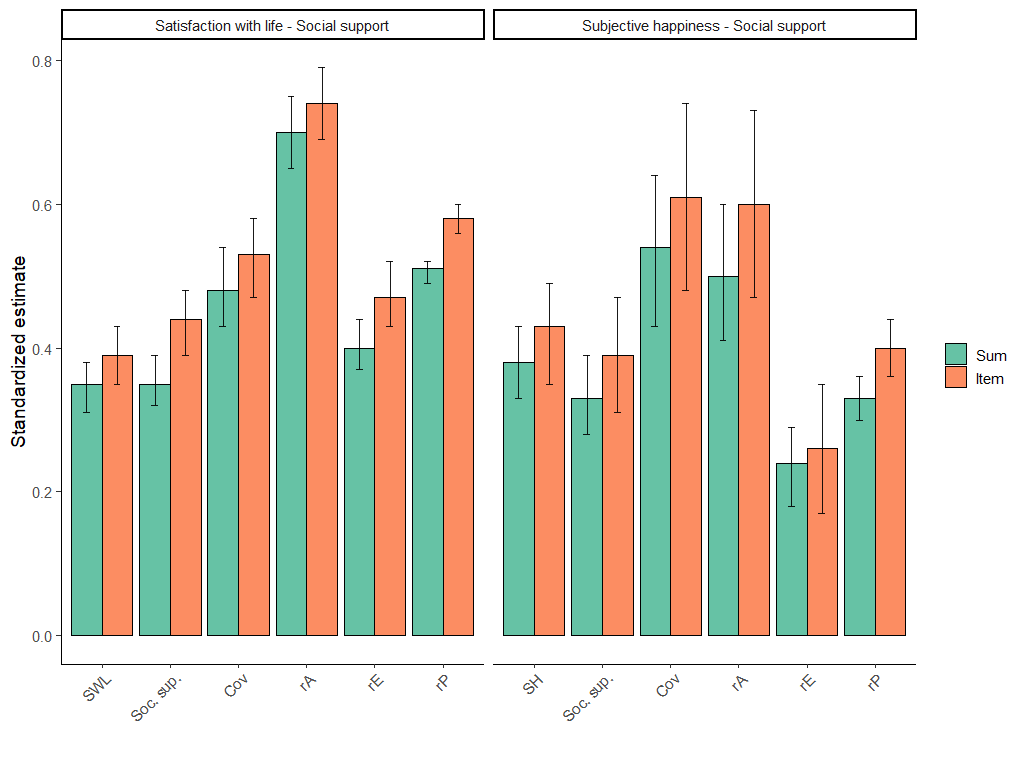


Figure S10. Standardized estimates from bivariate variance decomposition models. Values for SWL, SH, and Soc. sup. indicate heritability (*h*^2^) estimates, Cov represents the bivariate heritability estimate. SWL = Satisfaction with life, SH = subjective happiness, Soc. sup. = social support, Cov = covariance, rA = genetic correlation, rE = environmental correlation, rP = phenotypic correlation.
